# Direct Binding of Ledipasvir to HCV NS5A: Mechanism of Resistance to an HCV Antiviral Agent

**DOI:** 10.1371/journal.pone.0122844

**Published:** 2015-04-09

**Authors:** Hyock Joo Kwon, Weimei Xing, Katie Chan, Anita Niedziela-Majka, Katherine M. Brendza, Thorsten Kirschberg, Darryl Kato, John O. Link, Guofeng Cheng, Xiaohong Liu, Roman Sakowicz

**Affiliations:** Gilead Sciences, Inc., Foster City, California, United States of America; Scripps Research Institute, UNITED STATES

## Abstract

Ledipasvir, a direct acting antiviral agent (DAA) targeting the Hepatitis C Virus NS5A protein, exhibits picomolar activity in replicon cells. While its mechanism of action is unclear, mutations that confer resistance to ledipasvir in HCV replicon cells are located in NS5A, suggesting that NS5A is the direct target of ledipasvir. To date co-precipitation and cross-linking experiments in replicon or NS5A transfected cells have not conclusively shown a direct, specific interaction between NS5A and ledipasvir. Using recombinant, full length NS5A, we show that ledipasvir binds directly, with high affinity and specificity, to NS5A. Ledipasvir binding to recombinant NS5A is saturable with a dissociation constant in the low nanomolar range. A mutant form of NS5A (Y93H) that confers resistance to ledipasvir shows diminished binding to ledipasvir. The current study shows that ledipasvir inhibits NS5A through direct binding and that resistance to ledipasvir is the result of a reduction in binding affinity to NS5A mutants.

## Introduction

Hepatitis C Virus (HCV) infection is a leading cause of liver disease and hepatic cancer. An estimated 170 million individuals worldwide are infected with HCV [1]. HCV is a positive strand RNA virus and a member of the *Flaviviridae* family. The HCV genome encodes a polyprotein of ~3000 amino acids. The polyprotein is proteolytically cleaved by host and viral proteases to yield 10 proteins (3 structural proteins: core, E1, E2 and 7 non-structural proteins: p7, NS2, NS3, NS4A, NS4B, NS5A, NS5B) that are responsible for viral replication and assembly [2]. NS3-5B form a membrane associated complex that is responsible for replication of the HCV genome. Several direct acting antiviral (DAA) agents have been approved for use in patients with HCV, including the NS3/NS4A protease inhibitors telaprevir, boceprevir, and simeprevir and the NS5B polymerase inhibitor sofosbuvir [3]. Recently a new class of DAAs, that includes ledipasvir (LDV) and daclatasvir (DCV), has been identified that target NS5A [4, 5]. Treatment of patients with NS5A DAAs results in a rapid decline of viral load levels and it has been postulated that the rapid decline is the result of inhibition of RNA replication, virus assembly, and secretion [6–10].

NS5A is a phosphorylated protein [11] that is essential for viral replication, assembly, and secretion. NS5A has no known enzymatic activity, but interacts with other HCV proteins and numerous cellular factors (e.g. PKR, ApoA1) [12, 13]. *In vitro*, NS5A has been reported to bind single stranded RNAs, including the HCV 3’ untranslated region (UTR) positive-strand RNA [14–16]. NS5A is a 447 amino acid, membrane bound, zinc-binding protein. It contains an N-terminal amphipathic helix that anchors it to cellular membranes, followed by three hydrophilic domains [17]. Crystal structures of domain 1, which contains the zinc-binding motif, have revealed different dimeric structures [18–20]. Solution studies of domain 1 suggest it is monomeric [20], but a recent study performed in cell culture demonstrated intermolecular interactions between NS5A molecules [21].

NS5A has been implicated as the direct target of LDV and other NS5A DAAs. Drug associated resistance mutations (e.g. Y93H, L31V) map to NS5A-domain 1 [4, 22, 23]. Evidence from co-precipitation and cross-linking experiments in replicon cells using biotin or azide labeled inhibitors suggested a potential interaction between NS5A and DAAs [4, 24]. Finally, treatment of cells expressing the HCV replication complex with DCV resulted in a pronounced relocalization of NS5A to lipid droplets [25, 26]. Although these data show that NS5A is affected by LDV, DCV or other DAAs, they could not determine whether this was a result of specific binding to NS5A.

In the current study, we show that LDV binds directly to NS5A, but not the resistant Y93H mutant of NS5A. Binding of LDV could be competed with DCV, indicating that these two inhibitors bind in a specific manner to the same site on NS5A. These results define NS5A as the direct target of LDV and reveal the mechanism of resistance against these DAAs is due to reduced binding affinity to NS5A.

## Results

### Purification and Characterization of NS5A

**Fig 1:**
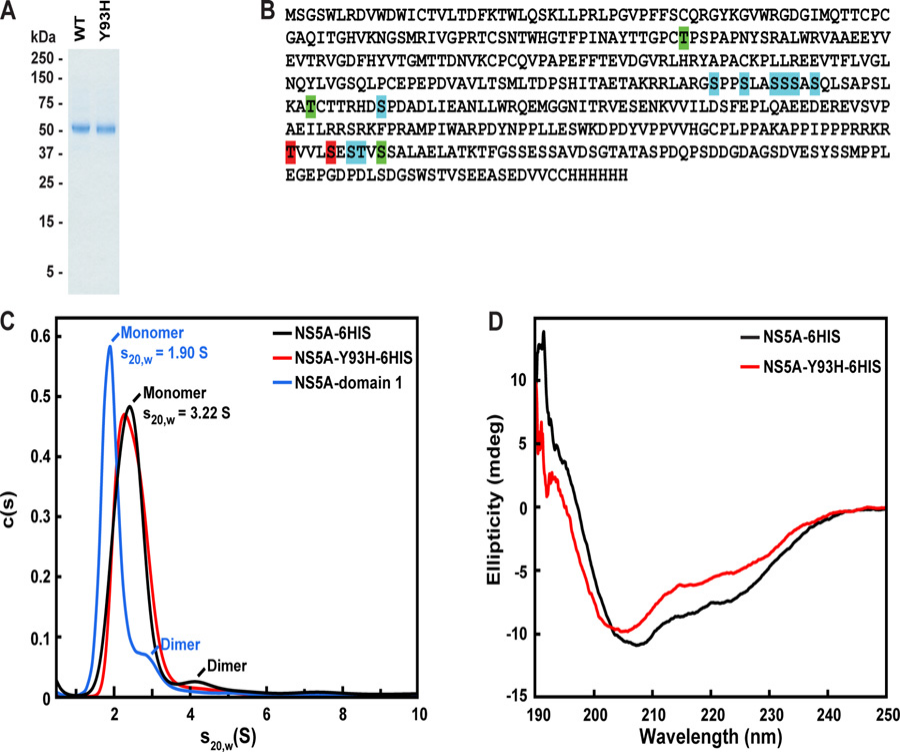
Characterization of purified NS5A. **(A)** Coomassie staining of purified proteins. Recombinant His-tagged proteins were purified as described in Materials and Methods. 1 μg of NS5A-6HIS and NS5A-Y93H-6HIS were subjected to SDS-PAGE and the protein bands were visualized by Coomassie Blue stain. Molecular weights for protein standards are indicated. **(B)** Phosphorylation sites determined by mass spectrometry of purified NS5A-6HIS and NS5A-Y93H-6HIS. Phosphorylation sites identified in NS5A-6HIS are shaded green; sites identified in NS5A-Y93H-6HIS are shaded red; and sites identified in both proteins are shaded cyan. **(C)** Analytical ultracentrifugation analysis of NS5A-6HIS, NS5A-Y93H-6HIS, and NS5A-domain 1. For NS5A-6HIS and NS5A-Y93H-6HIS, analytical ultracentrifugation was performed in 25 mM Tris pH 7.5, 150 mM NaCl, 0.01% NaN_3_, 0.5 mM TCEP, 2 μg/ml Leupeptin, 0.02% C12E8, and 21.1% (v/v) D_2_O. Sedimentation velocity analysis was performed at 42,000 rpm for two protein concentrations (3.3 and 6.6 μM). For NS5A-domain 1, analytical ultracentrifugation was performed in 25 mM Tris pH 8.0, 250 mM NaCl, 10% glycerol, and 0.5% DMSO. Sedimentation velocity analysis was performed at 48,000 rpm for 4 protein concentrations (7.5, 12, 15, 30 μM). Representative traces are shown for each protein. **(D)** Circular dichroism spectroscopy of NS5A-6HIS and NS5A-Y93H-6HIS. Circular dichroism measurements of NS5A-6HIS or NS5A-Y93H-6HIS (10 μM) were carried out at 20°C. The average of 3 measurements is shown.

A recombinant form of full length NS5A (genotype 1b, Con 1), containing the N-terminal amphipathic helix and a C-terminal His tag (NS5A-6HIS), was produced in SF9 cells using a baculovirus vector. The membrane bound protein was solubilized with the detergent C12E8 and purified to homogeneity using Ni affinity, ion exchange, and size exclusion chromatography. The identity and purity of NS5A-6HIS was confirmed by western blot and mass spectrometry (data not shown) and judged to be >90% pure by SDS-PAGE (Fig 1A). The Y93H mutant version of NS5A (NS5A-Y93H-6HIS) was expressed and purified in a similar manner.

We analyzed NS5A-6HIS and NS5A-Y93H-6HIS by mass spectrometry to determine the phosphorylation status of the proteins (Fig 1B). NS5A-6HIS or NS5A-Y93H-6HIS was digested with Lys-C and Trypsin and the peptide mixture was analyzed using accurate-mass nano-LC-MS/MS. Several phosphorylation sites were identified, including previously identified sites S222, S225, S229, and S232 [27].

We further characterized NS5A-6HIS and NS5A-Y93H-6HIS in C12E8 micelles using analytical ultracentrifugation and circular dichroism (CD) spectroscopy. The calculated molecular weight of NS5A-6HIS is 49.5 kD and that of a C12E8 micelle is ~65 kD. We carried out sedimentation velocity and equilibrium measurements using a H_2_O/D_2_O ratio that matched the density of the detergent molecules [28]. According to sedimentation velocity and equilibrium measurements, detergent solubilized NS5A-6HIS exists primarily as a monomer (80%), with a small percentage of dimers (6%) and higher order oligomers (14%) (Fig 1C). NS5A-Y93H contained a slightly lower amount of dimer (3%) and higher order oligomer (7%). In comparison, analysis of NS5A-domain 1 showed a similar ratio of monomer:dimer:oligomer to NS5A-6HIS, consistent with previous studies showing that NS5A-domain 1 is predominately monomeric in solution [20]. Fig 1D shows the far UV circular dichroism (CD) spectrum of NS5A-6HIS and NS5A-Y93H-6HIS in C12E8 micelles. NS5A-6HIS showed characteristics of a mixed alpha/beta structure. The CD spectrum of NS5A-Y93H-6HIS was similar to wild-type protein.

### Specific binding of LDV to NS5A

**Fig 2:**
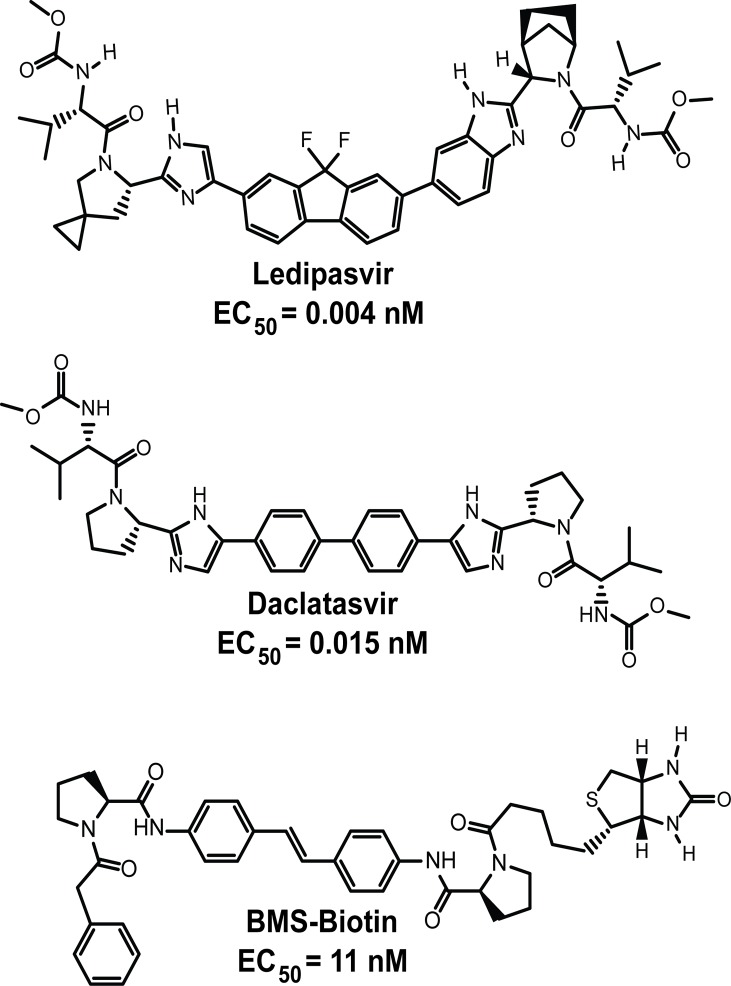
Structures of NS5A inhibitors. EC_50_ represents the 50% effective inhibitory concentration of HCV RNA replication in the Renilla luciferase replicon genotype 1b, Con1 cell line. EC_50_ for daclatasvir and BMS-Biotin (data not shown) were determined as previously described for ledipasvir [10].

**Fig 3:**
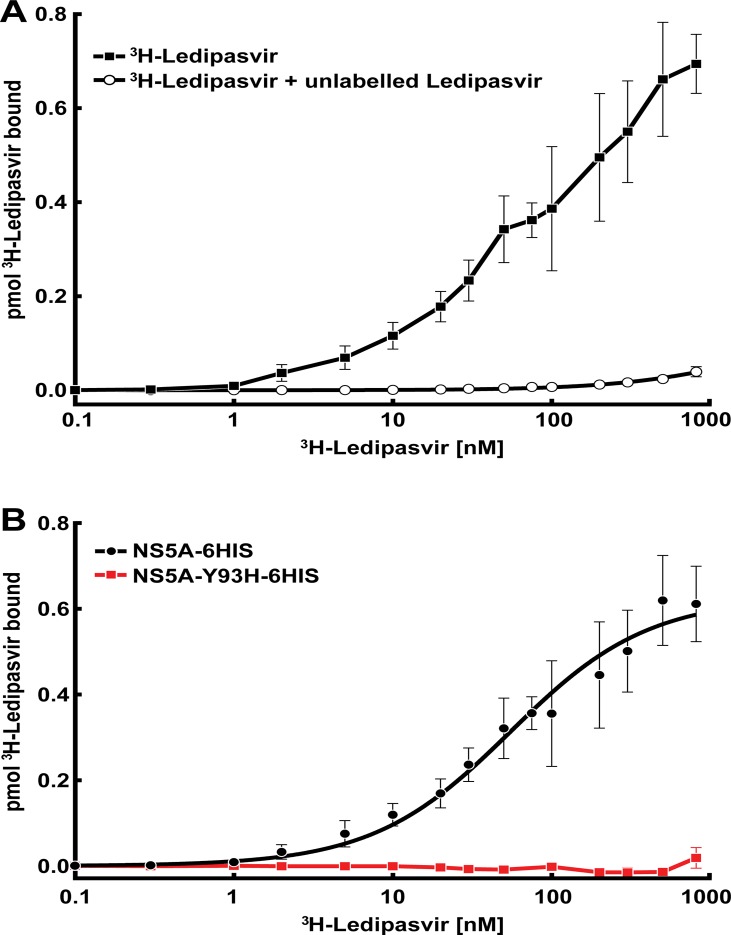
^3^H-LDV binding to NS5A-6HIS. **(A)** Each reaction, in a final volume of 200 μl, contained 50 nM of purified NS5A-6HIS and the indicated concentration of ^3^H-LDV in the absence (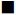) or presence (○) of unlabeled LDV. Bound ^3^H-LDV was measured as described in Materials and Methods. Each data point represents the average of 4–7 assays. **(B)** Specific binding of ^3^H-LDV to NS5A-6HIS (●) vs. NS5A-Y93H-6HIS (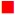). Specific binding was defined as the difference between the amount of ^3^H-LDV bound in the absence (total binding) and presence (non-specific binding) of unlabeled LDV. Each data point represents the average of at least 3 assays.

To monitor LDV binding to NS5A, we developed an *in vitro* radioligand binding assay (Figs 2 and 3). Tritium labeled LDV (^3^H-LDV) was incubated with recombinant NS5A-6HIS and the protein was bound to a Ni-NTA-agarose column. NS5A-6HIS was eluted and protein bound ^3^H-LDV was quantified by liquid scintillation counting (Fig 3A). Specific binding of ^3^H-LDV (defined by subtracting binding in the presence of 100 μM unlabeled LDV from the total binding) was saturable with a K_d_ = 58.9 ± 6.6 nM and a maximum specific binding B_max_ = 0.67 ± 0.2 pmol (for 10 pmol NS5A) (Fig 3B). At saturation, LDV bound to NS5A with a stoichiometry of one molecule of LDV per~15 NS5A monomers (7.5 dimers). This indicates that the amount of binding-competent NS5A was likely significantly lower than the nominal protein concentration. In contrast, binding of ^3^H-LDV to NS5A-Y93H-6HIS, a mutant form of NS5A resistant to drug inhibition [4, 29], was undetectable (Fig 3B). Given the solubility limits of LDV, we were unable to test binding of ^3^H-LDV above 10 μM and could not determine the K_d_ of LDV toward NS5A-Y93H.

**Fig 4:**
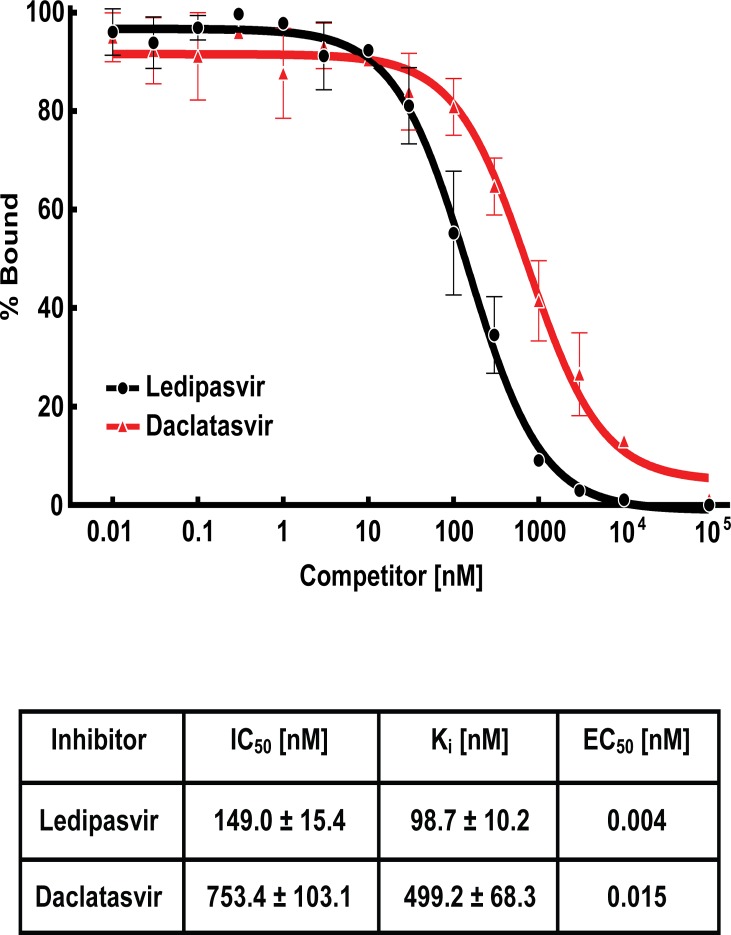
Competitive binding of ^3^H-LDV in the presence of unlabeled inhibitor. Each reaction, in a final volume of 200 μl, contained 50 nM NS5A-6HIS, 30 nM ^3^H-LDV and the indicated concentration of unlabeled LDV (●) or DCV (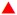). % Bound represents the amount of ^3^H-LDV bound relative to that in the control tube, which contained no unlabeled inhibitor. Each data point represents the average of at least 3 assays. K_i_ was calculated using the Cheng-Prusoff equation [30]. EC_50_ represents the 50% effective inhibitory concentration of HCV RNA replication in the Renilla luciferase genotype 1b, Con 1 replicon cell line.

We then carried out competitive binding studies to determine the relative affinity of the inhibitor DCV toward NS5A (Figs 2 and 4). Various concentrations of unlabeled LDV or DCV were incubated with a fixed concentration of ^3^H-LDV and NS5A and the ability of unlabeled inhibitor to compete for binding was determined (Fig 4). DCV was less potent than LDV with an IC_50_ = 753.4 vs. 149.0 nM respectively. These results indicate that DCV and LDV bind to the same site on NS5A.

### Co-Precipitation of Biotin-Labeled Inhibitors and NS5A from Replicon and Transfected Cells

**Fig 5:**
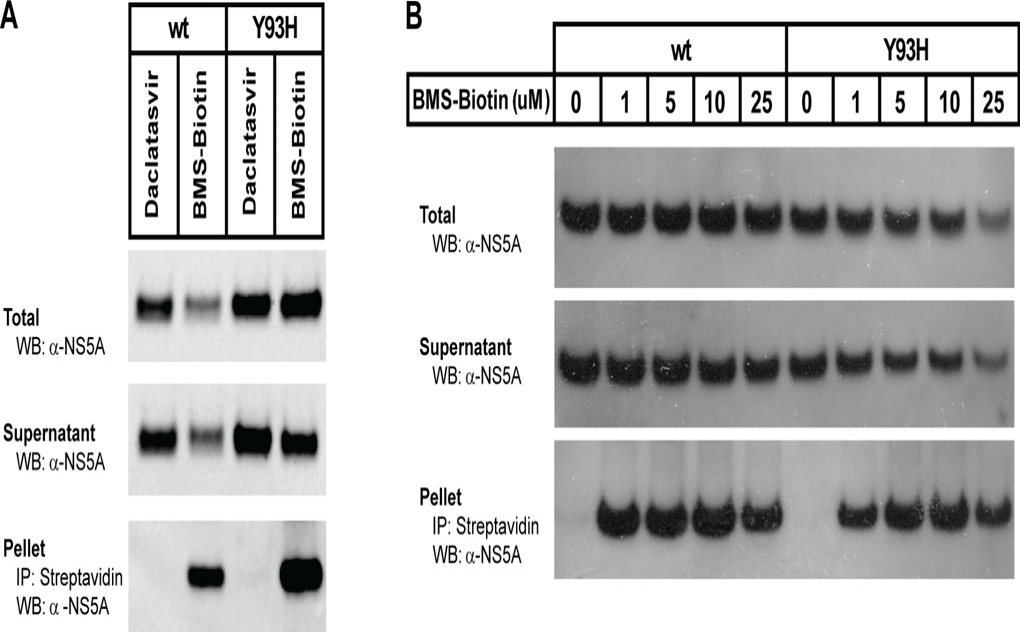
Co-precipitation of BMS-Biotin with NS5A and NS5A-Y93H. **(A)** Genotype 1b, Con1 replicon cells (wild type or harboring the Y93H mutant of NS5A) were treated with 1μM DCV or 25 μM BMS-Biotin and incubated for 24 hr. Cells were harvested and the membrane fraction was solubilized with C12E8. Solubilized proteins were incubated with streptavidin agarose beads and co-precipitated material was subjected to SDS-PAGE. NS5A was visualized by immunoblot analysis using an anti-NS5A antibody. **(B)** Huh7-lunet cells were transfected with pcDNA3-NS5A or pcDNA3-NS5A-Y93H. 24 hr post transfection, cells were treated with the indicated amount of BMS-Biotin, incubated for 42 hr and processed as in (A).

Gao et. al. showed previously that a biotin-labeled NS5A inhibitor (BMS-Biotin, Fig 2) could be used to co-precipitate NS5A from replicon cells [4]. Surprisingly, the Y93H resistant mutant of NS5A could also be co-precipitated with BMS-Biotin [4, 24]. We were able to reproduce the NS5A and NS5A-Y93H co-precipitation in genotype 1b, Con 1 replicon cells using the same biotin-labeled NS5A inhibitor (Fig 5A), but were unable to compete this interaction in replicon cells using non-biotin tagged NS5A inhibitors (data not shown).

Prolonged exposure of replicon cells to NS5A inhibitors leads to decreased replication and synthesis of NS5A, complicating analysis of NS5A in replicon cells treated with inhibitors. To circumvent this problem, we transfected Huh7-lunet cells with a plasmid containing NS5A or NS5A-Y93H under the control of a CMV promoter (pcDNA3-NS5A or pcDNA3-NS5A-Y93H). Transfected cells were treated with BMS-Biotin and membranes solubilized with detergent. The solubilized proteins were incubated with streptavidin-agarose and the co-precipitated NS5A protein was visualized by western blotting using an anti-NS5A antibody. NS5A and NS5A-Y93H both co-precipitated efficiently with BMS-Biotin (Fig 5B).

**Fig 6:**
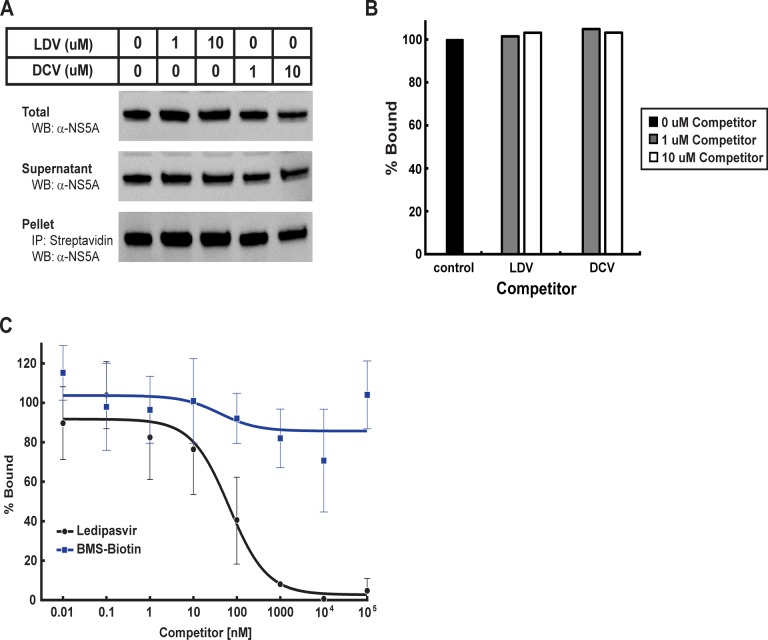
BMS-Biotin does not compete for LDV binding to NS5A. **(A)** Huh7-lunet cells were transfected with pTK-NS5A. 24 hr post transfection, cells were treated for 42 hr with a fixed concentration of BMS-Biotin and increasing concentrations of LDV or DCV. Cells were harvested and the membrane fraction was solubilized with C12E8. Solubilized proteins were incubated with streptavidin agarose beads and co-precipitated material was subjected to SDS-PAGE. NS5A was visualized by immunoblot analysis using an anti-NS5A antibody. **(B)** The relative intensity of pelleted NS5A, normalized to total NS5A, in (A) was quantified by densitometry. % Bound represents the amount of NS5A in the pellet relative to that in the control lane, which contained no competitor. **(C)**. Each reaction, in a final volume of 200 μl, contained 50 nM NS5A-6HIS, 30 nM ^3^H-LDV and the indicated concentration of unlabeled BMS-Biotin (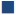) or LDV (●). % Bound represents the amount of ^3^H-LDV bound relative to that in the control tube, which contained no unlabeled inhibitor. Each data point represents the average of at least 3 assays.

We then attempted to perform competitive binding studies of BMS-Biotin and NS5A using LDV or DCV. In order to reduce the amount of BMS-Biotin needed to precipitate NS5A, we transfected Huh7-lunet cells with a plasmid containing NS5A under the control of the Herpes Simplex Virus thymidine kinase promoter (pTK-NS5A). The TK promoter resulted in ~100 fold less expression of NS5A (data not shown) compared to the CMV promoter. We treated the cells with a fixed concentration of BMS-Biotin and various amounts of LDV or DCV. We observed no significant decrease of co-precipitated NS5A in the presence of increasing concentrations of LDV or DCV (Fig 6A and 6B). These results recapitulate previous reports [4, 24], but were insufficient to conclude that the interaction with BMS-Biotin observed in these experiments are specific to NS5A.

### Competitive Binding of BMS-Biotin to NS5A

In order to reconcile the apparent binding of BMS-Biotin to NS5A observed in cell culture by co-precipitation, we performed *in vitro* competition studies to determine whether BMS-Biotin could compete for binding of ^3^H-LDV to NS5A. Various concentrations of BMS-Biotin were incubated with a fixed concentration of ^3^H-LDV and NS5A-6HIS. BMS-Biotin was unable to compete for binding of ^3^H-LDV to NS5A (Fig 6C). Given the solubility limits of BMS-Biotin, we were unable to perform competitive binding studies above 100 μM. At the highest concentration used in this assay, BMS-Biotin did not compete for LDV binding to NS5A.

## Discussion

LDV, a new class of DAAs that target NS5A, has recently emerged as a highly potent drug for the treatment of HCV infection [5]. While drug-associated resistance mutations have been identified in NS5A-domain 1, direct evidence of an interaction between this class of DAAs and NS5A has been elusive. The lack of direct observation of binding and the limited information regarding NS5A function has left the mechanism of resistance to LDV unclear. Indirect evidence from co-precipitation experiments using a Biotin labeled NS5A inhibitor (BMS-Biotin) have suggested a potential interaction with NS5A but could not elucidate the mechanism of resistance mutations. In the current study, we have developed an *in vitro* binding assay that directly measures LDV binding to NS5A. We show that LDV binds directly to NS5A at a specific, saturable site, providing direct evidence that NS5A is the target of LDV. We have also shown that, contrary to the results from co-precipitation experiments using Biotin labeled NS5A inhibitors, the NS5A drug resistance mutant Y93H has significantly reduced affinity toward LDV. Y93H is the primary NS5A drug resistance mutant selected in genotype 1b patients and replicon cells [10, 22]. Given the decreased antiviral potency of LDV against genotype 1b replicon cells harboring NS5A-Y93H (EC_50_ ~ 5300 pM), we expect the K_d_ of LDV to NS5A-Y93H is >10 μM and outside the detectable limit of our assay.

Biotin labeled inhibitors have been used in several studies to implicate NS5A as the direct target of NS5A DAAs. However, there was a lack of direct evidence that the interactions observed by co-precipitation were specific or relevant to LDV and other NS5A DAAs. BMS-Biotin did not discriminate between NS5A and the drug associated resistant mutant Y93H and unlabeled inhibitors could not compete for the interaction between BMS-Biotin and NS5A in transfected cells. Using our *in vitro* binding assay, we show that BMS-Biotin is unable to compete for binding of LDV to NS5A. Taken together, these results suggest that the interactions observed between BMS-Biotin and NS5A in cell culture may reflect binding to an alternative site or are the result of non-specific interactions with NS5A and NS5A-Y93H.

Ascher et. al. recently published a study showing DCV binding to bacterially expressed NS5A-domain 1 [31]. We were unable to observe binding of LDV to NS5A-domain 1 by a variety of biophysical techniques (data not shown). The full length NS5A used in this study contained the N-terminal amphipathic helix and was phosphorylated at several previously reported sites, in contrast to bacterially expressed NS5A-domain 1. Regions outside of domain 1 are important for NS5A function and are affected by NS5A inhibitors. The N-terminal amphipathic helix is important for HCV replication [32] and a number of drug resistant mutations are located at the N-terminus of NS5A-domain 1, adjacent to the amphipathic helix. Differential phosphorylation regulates NS5A function and affects its interaction with other proteins [33]. NS5A exists in basally phosphorylated and hyper-phosphorylated forms [11] and treatment of cells with NS5A inhibitors prevents hyper-phosphorylation [34]. The N-terminal amphipathic helix and phosphorylation state of NS5A may influence its structure, function, and binding to LDV and other NS5A DAAs. The differences observed *in vitro* for the binding of LDV to different forms of NS5A may reflect differences in experimental conditions, protein constructs, or post-translational modifications.

The concentration of NS5A in replicon cells has been estimated to be 0.6 to 600 nM [25] and 1 molecule of DCV can inhibit ~10,000 molecules of NS5A [12]. NS5A localization changes upon exposure to NS5A inhibitors and large aggregates containing both NS5A inhibitor and NS5A have been observed in replicon cells [21, 25]. The symmetric structure of DCV and the dimeric form of NS5A observed in crystal structures has led to the suggestion that one molecule of DCV binds to two molecules of NS5A [4, 18–20, 24]. Assuming two molecules of NS5A bind to one molecule of LDV, the stoichiometry observed in our *in vitro* binding assay suggests that LDV binds to the oligomeric fraction of NS5A. The current data support a model where LDV binds preferentially to higher order oligomers that while containing multiple LDV binding sites, only require a single site to be occupied to affect the oligomerization and function of NS5A. This could prevent reassociation of NS5A with the HCV replication complex and lead to the localization changes observed in cell culture. This model would explain why the affinity of LDV to purified NS5A was ~10000 fold weaker than the antiviral potency (EC_50_ = 4 pM). The effects of the oligomeric state, phosphorylation, genotypes, and other resistance mutations of NS5A on LDV binding are under current investigation.

## Materials and Methods

### Materials and Plasmids

^3^H-LDV (12.3 Ci/mmol) was obtained from Vitrax. LDV, Daclatasvir, and BMS-Biotin were synthesized by Gilead Sciences, Inc. and prepared in DMSO.

We generated a recombinant baculovirus encoding full length, wild-type NS5A containing a C-terminal 6HIS tag (NS5A-6HIS) by amplifying the nucleotide sequence for residues 1–447 of NS5A (genotype 1b, Con1) by PCR. The PCR fragment was digested with Stu1 and HindIII and ligated into pFastBac1 (Invitrogen). Baculovirus encoding the Y93H version of NS5A (NS5A-Y93H-6HIS) was generated by site-directed mutagenesis (Stratagene).

pcDNA3-NS5A, encoding full length NS5A, was generated by amplifying the nucleotide sequence for residues 1–447 of NS5A (genotype 1b, Con1) by PCR. The PCR fragment was digested with Xba1 and EcoRV and ligated into pcDNA3.1(-) (Invitrogen). pcDNA3-NS5A-Y93H was generated by site-directed mutagenesis (Stratagene). pTK-NS5A was constructed by digesting pcDNA3-NS5A with Xba1 and EcoRV to isolate a 1.5 kb fragment encoding NS5A. The 1.5 kb fragment was treated with T4 polymerase and ligated into pTK.

### Buffers and Media

Buffer A contained 25 mM Tris pH 7.5, 150 mM NaCl, 0.01% NaN_3_, 0.5 mM TCEP, 2 μg/ml Leupeptin, and 0.02% C12E8. Buffer B contained buffer A supplemented with 21.1% (v/v) D_2_O. Buffer C contained 25 mM Tris pH 7.5, 150 mM NaCl, 2 μg/ml Leupeptin, 0.5 mM TCEP, 0.01% NaN_3_, 0.001% C12E8, 1% ethanol, and 1% DMSO. Medium A contained Dulbelcco's modified Eagle Medium, 1X GlutaMAX-I (Invitrogen), 10% fetal bovine serum (Hyclone), 1 unit/ml penicillin (Invitrogen), 1 μg/ml streptomycin (Invitrogen), and 0.1 mM nonessential amino acids (Invitrogen).

### Protein Expression and Purification

NS5A-6HIS baculovirus was used to infect SF9 cells (Invitrogen) at 1.5x10^6^ cells/ml in ESF-921 medium (Expression Systems Limited). After incubation for 60 hr at 27°C, cells were pelleted by centrifugation, flash frozen in liquid nitrogen, and stored at -80°C. The frozen cell pellet from 1L cell culture was resuspended in 150 ml PBS containing 2 EDTA free protease inhibitor tablets (Roche) and 0.5 mM TCEP and lysed using a dounce homogenizer. Cellular membranes were isolated by centrifugation (100,000 x g pellet), resuspended in 150 ml buffer A supplemented with 1% C12E8, stirred for 2 hr at 4°C, and centrifuged at 100,000 x g for 1 hr at 4°C.

The supernatant, containing solubilized NS5A-6HIS, was applied to a 5ml Ni-NTA column (GE Healthcare) equilibrated in buffer A. The column was washed with 50 ml of buffer A containing 20 mM imidazole and NS5A-6HIS was eluted with 50 ml of buffer A containing 250 mM imidazole. Fractions containing NS5A-6HIS were pooled and concentrated to 10 ml in buffer A, and applied to an 8 ml monoQ column (GE Healthcare). NS5A-6HIS was eluted using a linear gradient from 150 mM to 1M NaCl. Fractions containing NS5A-6HIS were pooled, concentrated to 1 ml, and further purified by size exclusion chromatography using a 24 ml superose 6 column (GE Healthcare) equilibrated in buffer A. Protein was judged to be >90% pure by SDS-PAGE. Fractions containing NS5A-6HIS were concentrated, flash frozen in liquid nitrogen, and stored at -80°C prior to use. NS5A-Y93H-6HIS was expressed and purified as described above for wild type protein. NS5A-domain 1, encoding residues 25–215 of NS5A, was expressed and purified as previously described [20].

### Mass Spectrometry

Mass spectrometry of intact protein samples was performed with an Agilent 6210 Time of Flight Mass Spectrometer with a 4GHz upgrade and an Agilent 1200 Rapid Resolution HPLC. The samples were analyzed on an Agilent Zorbax 300 Extend C18 Rapid Resolution column at 70°C, using reverse phase chromatography with a gradient from 20 to 90% acetonitrile containing 0.1% formic acid. Data were analyzed using Agilent Masshunter B.06 Qualitative Analysis with the Bioconfirm upgrade.

1 nmol of NS5A protein in 1 mM TCEP, 20 mM iodoacetamide, 8M urea was incubated with 1 μg of Lys-C (Roche) and incubated for 4 hr at 37ºC. The digests were diluted 4 fold and incubated with 5 μg of trypsin for 12 hr at 37ºC. The digests were acidified with 0.1% formic acid and desalted with C-18 spin columns (Thermo Scientific). The eluted peptides were dried and resuspended in 10 μL of acetonitrile and 0.01% formic acid. The mixture of peptides was analyzed using accurate-mass nano-LC-MS/MS with an Agilent 6530 Quadrupole Time of Flight mass spectrometer. Chromatographic separation of peptides was performed with an Agilent 1260 Capillary/Nano-HPLC system coupled to a chip cube with a Phosphochip (Agilent Technologies) using a 0 to 90% acetonitrile gradient containing 0.1% formic acid. Data were analyzed by searching a customized protein database containing the sequence of NS5A using SpectrumMill (Agilent).

### Analytical Ultracentrifugation

Sedimentation velocity experiments of NS5A-6HIS and NS5A-Y93H-6HIS solubilized in C12E8 micelles were performed at 20°C in a ProteomeLab XL-A analytical ultracentrifuge. Samples of 400 μl of buffer B or various concentrations of NS5A-6HIS (3.3 and 6.6 μM) diluted in buffer B were loaded into a dual sector charcoal-filled epon centerpiece. The samples were centrifuged at 42,000 rpm in an An50-Ti rotor and sedimentation was monitored by absorbance spectrophotometry at a wavelength of 280 nm. Data were analyzed with the program SEDFIT, which generates a continuous c(s) distribution for the sedimenting species [35]. The program SEDNTERP was used to estimate the partial specific volume of the proteins as well as the density and viscosity of the buffer solutions. Sedimentation velocity experiments of NS5A-domain 1 were performed as described above for full-length protein with the following modifications. NS5A-domain 1 (7.5, 12, 15, and 30 μM) in 25 mM Tris pH 8.0, 250 mM NaCl, 10% glycerol, and 0.5% DMSO, was centrifuged at 48,000 rpm.

Sedimentation equilibrium of NS5A-6HIS solubilized in C12E8 micelles was performed at 20°C in a ProteomeLab XL-A analytical ultracentrifuge. The contribution of detergent micelles to the buoyant molecular mass of detergent-solubilized NS5A-6HIS was eliminated by adjusting the solvent density to equal that of the detergent [24]. The partial specific volume of C12E8 was assumed to be 0.973 ml/g. Sedimentation equilibrium data were collected using three protein concentrations (2, 4, and 8 μM) and three rotor speeds (14000, 17000, and 25000 rpm). Data were analyzed using a non-linear least squares model.

### Circular Dichroism Spectroscopy

Circular dichroism spectroscopy was carried out on a Jasco J-815 CD spectrometer using a 0.2 mm path length cuvette. Far-UV (190–250 nm) CD spectra of 10 μM NS5A-6HIS and NS5A-Y93H-6HIS were measured at 20°C and corrected for solvent (buffer A).

### ^3^H-LDV Binding Assay

^3^H-LDV (12.3 Ci/mmol) was prepared in ethanol and stored at -20°C prior to use. For binding reactions, 50 nM NS5A-6HIS was mixed with varying concentrations of ^3^H-LDV in the absence or presence of 100 μM of unlabeled LDV. Each reaction, in a final volume of 200 μl of buffer C, was incubated for 4 hr at room temperature and then loaded onto a 2-ml column packed with 0.25 ml of Ni-NTA-agarose beads. Each column was washed with 5 ml of buffer A and ^3^H-LDV bound protein was eluted with 1.5 ml of buffer A containing 300 mM imidazole. The amount of eluted ^3^H-LDV was quantified by liquid scintillation counting. Specific binding was defined as the difference between the amount of ^3^H-LDV bound in the absence (total binding) and presence (non-specific binding) of unlabeled LDV. Data were fit to a single site binding model to obtain the dissociation constant (K_d_) and maximum specific binding (B_max_): Bound = B_max_*[L]/(K_d_ + [L]), where [L] is the concentration of ^3^H-LDV.

For competition reactions, 50 nM NS5A-6HIS was mixed with a fixed concentration of ^3^H-LDV (30 nM) and varying concentrations of unlabeled LDV, DCV, or BMS-Biotin (in DMSO). Each reaction, in a final volume of 200 μl of buffer C, was incubated at room temperature for 4 hr and processed as described above. Data were fit to a single site competitive binding model to obtain the concentration of half-maximal binding (IC_50_): %Bound = B_min_ + (B_max_-B_min_)/(1 + [S]/IC_50_), where %Bound is the fraction of ^3^H-LDV bound relative to control with no competitor, B_min_ and B_max_ are parameters related to %Bound when fully inhibited and uninhibited respectively, and [S] is the concentration of competitor. The inhibitor dissociation constant (K_i_) was determined using the Cheng-Prusoff equation [30] and the K_d_ determined from the saturation binding experiment: K_i_ = IC_50_/(1 + [^3^H-LDV]/K_d_).

### Co-Precipitation of BMS-Biotin and NS5A from Replicon or Transfected Cells

On day 0, Huh7-lunet cells stably expressing the genotype 1b, Con 1 replicon or the genotype 1b, Con1 replicon containing mutant NS5A-Y93H [36] were plated in medium A at a density of 12x10^6^ cells per T175 flask and cultured at 37°C. On Day 2, cells were switched to medium A containing 1 μM DCV or 25 μM BMS-Biotin. After incubation for 24 hr, cells were washed with PBS, trypsinized, and harvested by centrifugation.

On day 0, Huh7-lunet cells were plated in Medium A without antibiotics at a density of 1x10^6^ cells per T75 flask and cultured at 37°C. On day 1, cells were transfected with the indicated plasmid using Lipofectamine LTX (Invitrogen). On day 2, cells were switched to medium containing BMS-Biotin and varying amounts of LDV or DCV. After incubation for 42 hr, cells were washed with PBS and harvested by centrifugation.

Cells were lysed by passage through a 27-gauge needle, and membranes were isolated by centrifugation (100,000 x g pellet). Membranes were solubilized with PBS containing 0.1% C12E8 and solubilized protein was isolated by centrifugation (100,000 x g supernatant). BMS-Biotin was precipitated from the soluble fraction by incubation with streptavidin-agarose beads (Pierce). The pelleted beads were washed and co-precipitated material was eluted with SDS loading buffer. Samples were subjected to SDS-PAGE and immunoblot analysis using an anti-NS5A monoclonal antibody (Apath LLC).
